# Cystathionine-γ-lyase expression is associated with mitochondrial respiration during sepsis-induced acute kidney injury in swine with atherosclerosis

**DOI:** 10.1186/s40635-018-0208-z

**Published:** 2018-10-20

**Authors:** Tamara Merz, Martin Wepler, Benedikt Nußbaum, Josef Vogt, Enrico Calzia, Rui Wang, Csaba Szabo, Peter Radermacher, Oscar McCook

**Affiliations:** 1 Institut für Anästhesiologische Pathophysiologie und Verfahrensentwicklung, Universitätsklinikum Ulm, Helmholtzstrasse 8-1, 89081 Ulm, Germany; 2 Klinik für Anästhesiologie, Universitätsklinikum Ulm, Ulm, Germany; 3 Department of Biology, Laurentian University, Sudbury, ON Canada; 4 Department of Anesthesiology, The University of Texas Medical Branch at Galveston, 601 Harborside Drive, Galveston, TX 77555 USA; 5 Department of Oncology, Microbiology and Immunology, Faculty of Science and Medicine, University of Fribourg, Fribourg, Switzerland

**Keywords:** Peroxisome proliferator-activated receptor gamma coactivator 1-α, Barrier dysfunction, Oxidative stress, Co-morbidity, Glucose utilization, Hyperlactatemia

## Abstract

**Background:**

Sepsis is associated with disturbed glucose metabolism and reduced mitochondrial activity and biogenesis, ultimately leading to multiple organ dysfunction, e.g., acute kidney injury (AKI). Cystathionine-γ-lyase (CSE), the major cardiovascular source of endogenous H_2_S release, is implicated in the regulation of glucose metabolism and mitochondrial activity through a PGC1α-dependent mechanism, and critical for kidney function. Atherosclerosis is associated with mitochondrial dysfunction and reduced CSE expression. Thus, the aim of this post hoc study was to test the hypothesis whether there is an interplay between CSE expression and kidney dysfunction, mitochondrial activity, and oxidative/nitrosative stress in porcine septic AKI with underlying coronary artery disease.

**Methods:**

This study is a *post hoc* analysis of material from anesthetized and instrumented swine with a high fat diet-induced hypercholesterolemia and atherosclerosis undergoing faecal peritonitis-induced septic shock or sham procedure and intensive care (comprising fluid resuscitation and continuous i.v. noradrenaline (NoA) infusion) for 24 h. Glucose metabolism was quantified from blood ^13^C_6_-glucose and expiratory ^13^CO_2_/^12^CO_2_ isotope enrichment during ^13^C_6_-glucose infusion. Mitochondrial activity was determined by high-resolution respirometry. CSE and PGC1α expression, as well as nitrotyrosine formation and albumin extravasation, were quantified by immunohistochemistry of formalin-fixed kidney paraffin sections.

**Results:**

Sepsis was associated with lactic acidosis (*p* = 0.004) and AKI (50% fall of creatinine clearance (CrCl), *p* = 0.019). While both whole-body glucose production (*p* = 0.004) and oxidation (*p* = 0.006) were increased, kidney tissue mitochondrial respiration was reduced (*p* = 0.028), coinciding with decreased CSE (*p* = 0.003) and PGC1α (*p* = 0.003) expression. Albumin extravasation (*p* = 0.011) and nitrotyrosine formation (*p* = 0.008) were increased in septic kidneys.

**Conclusions:**

Sepsis-induced AKI is associated with disturbed mitochondrial respiration and biogenesis, which may be aggravated by oxidative and nitrosative stress. Our results confirm previous data in murine septic shock and porcine hemorrhage and resuscitation on the crucial role of CSE for barrier integrity and kidney function.

**Electronic supplementary material:**

The online version of this article (10.1186/s40635-018-0208-z) contains supplementary material, which is available to authorized users.

## Background

Septic acute kidney injury (AKI) is characterized by an acute decrease in renal function coinciding with relatively normal histologic morphology [1–5]. The severity of septic AKI is related to vascular leakage [1], derangements in glucose metabolism [6], and reduced mitochondrial activity [7, 8]. Hydrogen sulfide (H_2_S), an important “gaseous mediator,” has been implicated in all the abovementioned: kidney function, vascular regulation, barrier function, glycemic control, and the mitochondria. Cystathionine-γ-lyase (CSE), the major enzymatic source for endogenous H_2_S, is constitutively expressed in the kidney and has been shown to be critical for kidney function [9, 10] as its “abundant” expression is associated with improved glomerular filtration in human transplant patients [11]. We recently demonstrated that CSE expression correlated with maintained creatinine clearance (CrCl) and reduced vascular leakage in resuscitated murine polymicrobial septic shock [1].

CSE has also been shown to play a role in regulating glucose metabolism and mitochondrial function through a PGC1α-dependent mechanism [8, 12]. CSE^−/−^ mice displayed reduced gluconeogenesis, which was reversed by exogenous administration of H_2_S, through the upregulation of PGC1α [12]. In addition, we recently demonstrated the inverse relationship, suggesting a regulatory loop; in hyperglycemic septic mice, CSE protein expression was downregulated concomitant with reduced PGC1α levels and mitochondrial respiratory activity in both coupled (maximum oxidative phosphorylation (OxPhos)) and uncoupled (maximum capacity of the electron transfer system) states [8]. Impaired renal mitochondrial function, through its production and release of free radicals, may further aggravate organ injury during sepsis [13]. H_2_S is a mitochondrial electron donor and a free radical scavenger [14] and has been demonstrated to attenuate AKI in pre-clinical ischemia/reperfusion models [9]. Its role in sepsis, however, is ambivalent: In rodent endotoxemia or sepsis, H_2_S was reported to attenuate [15, 16] or aggravate [17] kidney dysfunction and/or injury.

Atherosclerosis is a common confounding factor in the management of sepsis, increasing mortality [18], which is often not incorporated by the use of naive/young animal models [9]. We recently reported that atherosclerotic pigs with coronary artery disease subjected to sepsis developed impaired cardiac function, which coincided with decreased CSE expression and increased nitrotyrosine formation [19, 20]. Thus, the aim of this study was to test the hypothesis whether there is an interplay between CSE expression and kidney dysfunction, mitochondrial activity, and oxidative/nitrosative stress in porcine septic AKI with underlying co-morbidity. The used swine strain, “familial hypercholesterolemia Bretoncelles Meishan” (FBM), is characterized by coronary artery disease due to an atherogenic diet. FBM animals are known to exhibit significantly higher cholesterol levels compared to healthy swine of the same age [21–23] and present the biomarker pattern of hypercholesterolemia: increased oxidative stress and lower blood levels of nitric oxide (NO) metabolites [23] reminiscent of patients with hypercholesterolemia-induced atherosclerosis [24]. In atherosclerosis, CSE has been shown to play an important role: its downregulation is associated with hypertension, cardiovascular pathology, coronary artery disease, and chronic kidney disease [20, 25]. Data reported are a post hoc analysis of material available from a previous study [26].

## Methods

The study was approved by the University of Ulm Animal Care Committee and the Federal Authorities for Animal Research. The experiments were performed in adherence to the National Institute of Health Guidelines on the Use of Laboratory Animals and the European Union “Directive 2010/63/EU on the protection of animals used for scientific purposes” and authorized by the federal authorities for animal research of the Regierungspräsidium Tübingen (approved animal experimentation number: 1024), Baden-Württemberg, Germany, and the Animal Care Committee of the University of Ulm, Baden-Württemberg, Germany. This is a post hoc study performed on available material from the vehicle-treated group of a previous study [26] and unpublished sham-operated animals studied simultaneously under the same protocol. The underlying atherosclerosis in the pig strain has previously been characterized in coronary vascular sections by our group [19, 20]. Briefly, male castrated FBM (age 15–30 months, 69 kg (65–73 kg)) with a high-fat diet-induced hypercholesterolemia and atherosclerosis [21] underwent polymicrobial septic shock (*n* = 8) induced by inoculation of autologous feces into the abdominal cavity, or sham procedure, i.e., abdominal saline injection (*n* = 5), and subsequently received intensive care therapy for 24 h. Anesthesia and surgical instrumentation have been previously described in detail [26, 27]. In brief, for all animals, anesthesia was induced with propofol and ketamine to allow endotracheal intubation and was maintained thereafter with continuous i.v. pentobarbitone and pancuronium and intermittent buprenorphine. Ventilator settings were fraction of inspired O_2_ (FiO_2_) 0.35, positive end-expiratory pressure (PEEP) 10 cmH_2_O, tidal volume 8 mL/kg, respiratory rate 10 to 12 breaths/min adjusted to maintain arterial PCO_2_ = 35 to 40 mmHg, inspiratory (I)/expiratory (E) ratio 1:1.5, peak airway pressure < 40 cmH_2_O, and modified to I/E ratio 1:1, and PEEP 12 or 15 cmH_2_O if the ratio of arterial O_2_ partial pressure (PaO_2_)/FiO_2_ is < 300 or < 200 mmHg, respectively [26]. The septic and sham pigs had the right jugular vein and carotid artery exposed for the insertion of a central venous catheter sheath and the placement of a balloon-tipped pulmonary artery catheter to measure central venous pressure (CVP) and a thermistor-tipped arterial catheter for blood pressure (mean arterial pressure (MAP)) recording and transpulmonary single indicator thermodilution–cardiac output measurement. Ringer’s solution was continuously infused as maintenance fluid (10 mL/(kg h)) [20].

### Experimental protocol

Atherogenic diet (1 kg daily, 1.5% cholesterol, 20% bacon fat) was fed for at least 9 months prior to the experiments. Post anesthesia and surgical instrumentation, the supernatant (3 mL/kg) of 1.0 g/kg autologous feces incubated in 500 mL 0.9% saline for 12 h at 38 °C, or saline only as sham procedure, was injected into the abdominal cavity via the abdominal drainage tubes. Hydroxyethyl starch (10 mL/(kg h)), 5 mL/(kg h) if CVP or PAOP was > 18 mmHg, allowed maintaining hyperdynamic hemodynamics. If necessary, norepinephrine was infused and titrated to maintain MAP at baseline values (not further increased if the heart rate was ≥ 160 beats/min to avoid tachycardia-induced myocardial ischemia) [26]. Twenty-four hours after the induction of fecal peritonitis, anesthesia was further deepened and animals were sacrificed with potassium chloride [20]. At the end of the experiment, the total amount of NoA given to the animal was documented. This amount was then normalized to the duration of the experiment and body weight of the animal to be able to compare averaged NoA infusion rates between the individual animals.

### Measurements and calculations

Hemodynamics, systemic and pulmonary gas exchange (arterial and mixed venous blood gases), creatinine, and renal glucose, lactate, and hemoglobin concentrations have been described previously in the original publication [26]. Using a steady-state approach, endogenous glucose production was calculated as the difference between the rate of appearance of stable, non-radioactively labeled 1,2,3,4,5,6-^13^C_6_-glucose during continuous intravenous isotope infusion minus the exogenous glucose infusion rate after gas chromatography-mass spectrometry assessment of plasma isotope enrichment. Direct, aerobic glucose oxidation was derived from the mixed expiratory ^13^CO_2_ isotope enrichment measured using non-dispersive infrared spectrometry and corrected for the plasma isotope ratio and CO_2_ production [23, 28]. Urinary and renal blood creatinine levels were analyzed to calculate creatinine clearance; renal venous NGAL, TNFα, and IL-6 levels were assessed as previously described [23, 26].

### Histopathology and immunohistochemistry

For this experiment on the kidney, we selected the pig in contrast to the mouse, because pigs, monkeys, and humans share multilobular, multipapillary kidneys in contrast to rodent kidneys, which are unilobular and unipapillary. Furthermore, in rodents, the urine empties directly into the renal pelvis, whereas in pigs and humans, urine empties into a branched caliceal network that distributes to the renal pelvis. The intricate system of interlobar and segmented arteries, that provides blood flow to numerous kidney lobes in humans and pigs, is not present in rodents and dogs because they do not have the multiple medullary pyramids [29]. As a consequence, kidney ischemic injury in rodents leads to extensive necrosis of proximal tubules. In humans, in contrast, “frank tubular necrosis” is infrequent, less pronounced, and only patchy if present at all [9, 30].

Post-mortem, pyramid-shaped kidney samples comprising the kidney cortex, medulla, and renal papilla were fixed in formalin (fixation identical for all samples), dehydrated, and embedded in paraffin blocks. Paraffin sections (3–5 μm) were cut, deparaffinized in xylene, and rehydrated with a graded series of ethanol to deionized water. Histopathological examination of hematoxylin–eosin-stained specimens was performed by an experienced pathologist blinded for the sample grouping [26]. Histopathological alterations were analyzed for the degree of “glomerular tubularization,” dilatation of Bowman’s space, and swelling of Bowman’s capsule, cellular edema of the proximal tubule, distal tubular dilatation and elongation, tubular protein cylinders, and tubular necrosis as described in detail previously [26].

For immunohistochemistry, the slide sections containing sham and septic tissue were analyzed concurrently and included both negative and positive controls. After heat-induced antigen retrieval in citrate pH 6, the slides were blocked with 10% normal sera (Jackson ImmunoResearch) before incubating in primary antibody (1° ab, anti-nitrotyrosine (Millipore)), CSE: anti-CTH (Abnova), anti-PGC1α (Novus), and anti-pig albumin (Abcam)). Primary antibody detection was performed by Dako REAL detection system (anti-mouse, anti-rabbit; alkaline phosphatase conjugated) and visualized with red chromogen (Dako REAL; Dako) followed by counterstaining with hematoxylin (Sigma). The slides were visualized using a Zeiss Axio Imager A1 microscope with a × 10 objective. Quantification for intensity was performed on multiple 800,000-μm^2^ sections using the AxioVision 4.8 software (Zeiss) [10]. Data are presented as densitometric sum red [20]. The obtained densitometric values for CSE were compared and correlated (*p* = 0.001) with the values obtained by western blotting (data shown in Additional file 1). It is well established in the literature that densitometric analysis of colorimetric immunohistochemical staining is as acceptable a method as western blotting for protein measurement [31–33]. In contrast to western blot, the IHC evaluation of the tissue allows identification of the physical topography and protein expression in different cell types.

### Mitochondrial respiration

Mitochondrial respiratory activity was determined via high-resolution respirometry with a Clark-electrode-based system (Oxygraph 2k, OROBOROS Instruments Corp., Innsbruck, Austria) as described previously [34, 35]. Fresh postmortem kidney samples were collected in Custodiol (Franz Köhler Chemie) and mechanically homogenized in respiration medium (MIR05; 0.5 mM EGTA, 3 mM MgCl_2_·6H_2_O, 60 mM lactobionic acid, 20 mM taurine, 10 mM KH_2_PO_4_, 20 mM HEPES, 110 mM sucrose, and 1 g/L bovine serum albumin), and 2 mg of tissue was added to the Oxygraph chamber. The chambers were oxygenated to an O_2_ concentration of 400 μM. By the addition of a defined sequence of substrates and inhibitors, various states of mitochondrial function could be assessed. Ten micromolar cytochrome c was added to reactivate mitochondria after homogenization. Complex I activity was determined after the addition of 10 mM pyruvate and glutamate, and 5 mM malate and ADP. Maximum OxPhos was evaluated after subsequent addition of 0.5 mM octanoyl–carnitine and 10 mM succinate; leak compensation was assessed after inhibition of the ATP-synthase by 2.5 μM oligomycin, followed by stepwise titration of the uncoupling agent carbonyl cyanide-4-(trifluoromethoxy) phenylhydrazone (FCCP, final concentration 1.5 μM) to reach maximum respiratory activity of the electron transfer system (ETS) in the uncoupled state. The activity of complex II was determined in the uncoupled state by the addition of 0.5 μM rotenone, an inhibitor of complex I. The measurement was finished after the addition of 5 μM of the complex III inhibitor antimycin A [8].

### Statistical analysis

Data are presented as median (quartiles). Data on physiology and renal blood gas analysis were analyzed with a two-way ANOVA and post hoc Tukey test for multiple comparisons. All other group differences were analyzed with the Mann–Whitney rank sum test after exclusion of normal distribution using the Kolmogorov–Smirnov test. Quantitative relations of pooled data sets from all experimental groups, between noradrenaline infusion and creatinine clearance, between noradrenaline infusion and mitochondrial activity, and between mitochondrial activity and creatinine clearance, were determined with Spearman’s coefficient for non-linear relationships, and the data were fitted to an exponential decay or growth according to the general equation *f*(*x*) = *a* · *e*^±*bx*^. Correlations between CSE expression and PGC1α expression, albumin extravasation, nitrotyrosine formation, and mitochondrial activity as well as correlations between nitrotyrosine and mitochondrial activity were evaluated by measuring Pearson’s coefficient of correlation for linear relationships. A non-linear model was used whenever a linear model was not statistically significant and/or a markedly higher coefficient of correlation suggested better fitting of the non-linear model. Due to the post hoc character of the study, the complexity of the experiment, and the high amount of parameters evaluated, unfortunately, not all values were available for all the animals. The actual *n* for the individual data sets is given in the respective figure legend.

## Results

### Physiological data

**Fig. 1:**
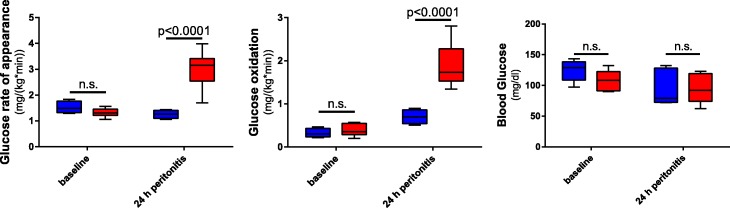
Glucose metabolism. Glucose rate of appearance (sham *n* = 4, sepsis *n* = 7) (left panel) indicates endogenous glucose production. Aerobic whole-body glucose oxidation (sham *n* = 4, sepsis *n* = 5) (middle panel). Renal venous blood glucose levels (sham *n* = 5, sepsis *n* = 8) (right panel). Blue boxes, sham group; red boxes, sepsis group. n.s. *p* > 0.05

Additional file 1: Table S1 displays systemic physiologic parameters. Norepinephrine requirements to maintain hemodynamics were significantly higher in the septic group (NoA *p* = 0.004). Furthermore, there was evidence that the septic pigs developed lactic acidosis, systemically (Additional file 1: Table S1), as well as on the kidney level (Additional file 1: Table S2). Inflammatory cytokines TNFα and IL6, determined from the renal venous blood, were elevated in sepsis (Additional file 1: Table S2). Septic pigs increased their glucose production and oxidation over time (see Fig. 1), whereas glucose metabolism did not change in the sham pigs. Blood glucose levels did not differ between the groups.

### Histopathology

**Fig. 2:**
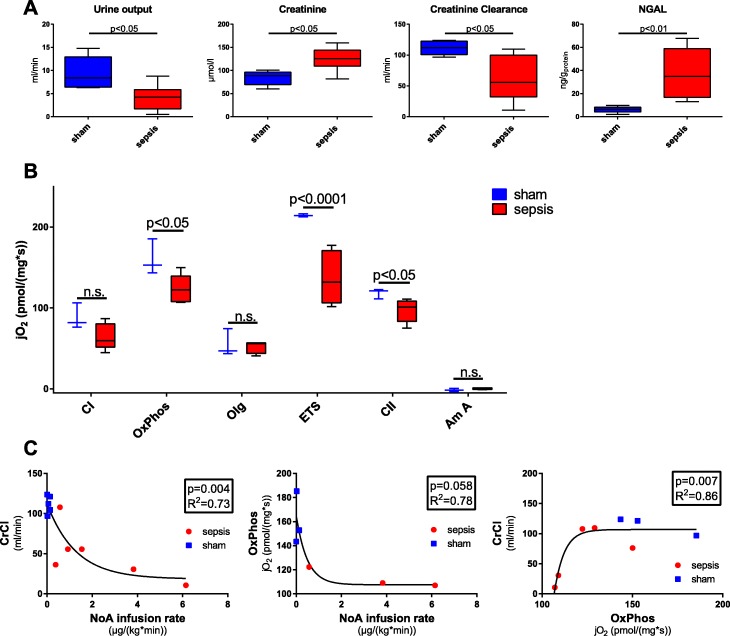
Parameters of kidney function. **a** Physiological parameters: urine output, plasma creatinine (Creatinine), creatinine clearance (CrCl), and renal venous neutrophil gelatinase-associated lipocalin (NGAL) (all values sham *n* = 5, sepsis *n* = 8). **b** Kidney mitochondrial respiratory activity (sham *n* = 3, sepsis *n* = 5). jO_2_, oxygen consumption; CI, mitochondrial respiratory activity in the coupled state stimulated with complex I substrates and ADP; OxPhos, maximum mitochondrial respiratory activity in the coupled state with complex I and II substrates, fatty acids, and ADP; Olg, leak respiration after inhibition of ATP-synthase with oligomycin; ETS, maximum mitochondrial respiratory activity after uncoupling with FCCP; CII, uncoupled mitochondrial respiratory activity linked to complex II after inhibition of complex I by rotenone. **c** Plotting of CrCl and mitochondrial activity to noradrenaline infusion (NoA). *p < 0.05; **p < 0.01; n.s. *p* > 0.05

Kidney histopathology in the septic animals showed only mild to moderate glomerular and tubular damage with very limited apoptosis and necrosis (see Table 1). Nevertheless, organ function was significantly impaired by sepsis (see Fig. 2a), as evinced by 50% reduced urine output (*p* = 0.011), 40% elevated plasma creatinine (*p* = 0.011), and, consequently, 50% reduced creatinine clearance (*p* = 0.019). Renal vein NGAL levels were significantly elevated in sepsis (see Fig. 2a).

**Table 1:** Kidney histopathology score

	Sham	Sepsis
% glomerular tubularization	2.5 (0.5; 7.5)	5.0 (0; 13.8)
Tubular apoptosis/necrosis	0	0
Protein cylinders	0 (0; 0)	1 (0; 1)
Dilatation of Bowman’s space	0 (0; 1)	2 (0; 3)

Glomerular tubularization, i.e., the herniation of proximal tubular epithelial cells into Bowman’s capsule along the luminal surface of the capsule, is reported as the number of glomeruli showing herniation of the tubular epithelium in % of all glomeruli analyzed; all other data are the mean values of the scores of the five random sections for each item analyzed. Data are given as median (interquartile range)

### Mitochondrial respiration

Impaired kidney function coincided with significantly reduced mitochondrial activity in the coupled as well as in the uncoupled state (see Fig. 2a), linked to the reduced activity of complex II. There was a trend towards stronger coupling and higher Olg respiration in the mitochondria from the septic kidney (OxPhos/ETS 0.92 (0.82; 1.02) vs. 0.71 (0.68; 0.86), *p* = 0.14; Olg/ETS 0.36 (0.28; 0.53) vs. 0.22 (0.20; 0.34), *p* = 0.14).

Plotting CrCl and OxPhos as functions of noradrenaline infusion rates revealed a significant inverse relationship between kidney function and mitochondrial activity to catecholamine requirements, respectively. Consequently, CrCl correlated with mitochondrial respiratory function.

### Protein expression

**Fig. 3:**
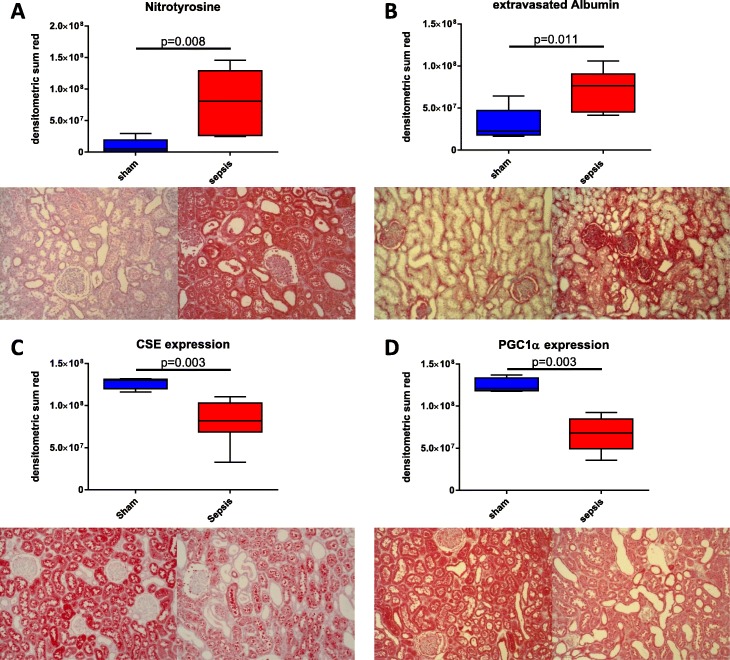
Kidney specimens stained for nitrotyrosine formation (sham *n* = 5, sepsis *n* = 8) (**a**), albumin extravasation (sham *n* = 5, sepsis *n* = 8) (**b**), CSE expression (sham *n* = 5, sepsis *n* = 7) (**c**), and PGC1α expression (sham *n* = 5, sepsis *n* = 8) (**d**). Top quantitative densitometric sum (red), bottom exemplary pictures for sham and sepsis, respectively, in × 10 magnification

**Fig. 4:**
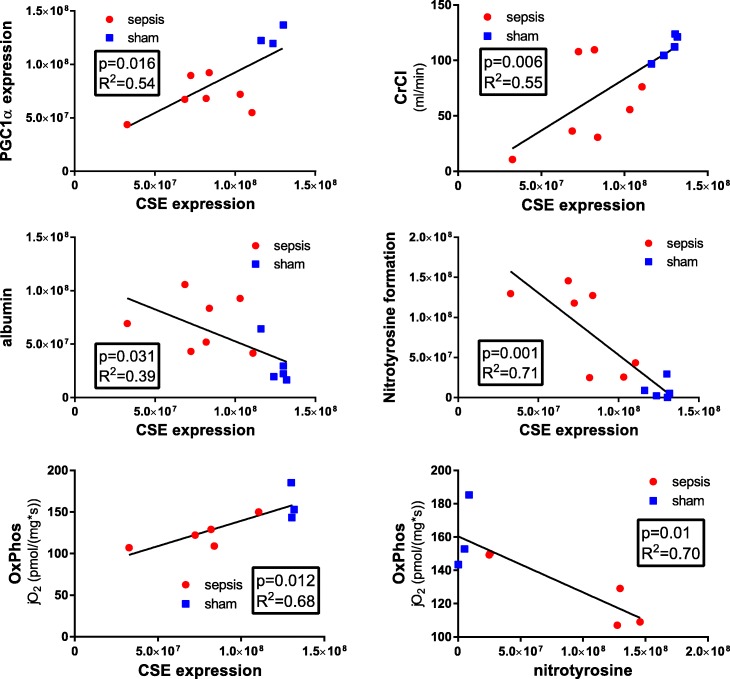
Mathematical correlations of PGC1α expression, kidney function (CrCl), albumin extravasation, nitrotyrosine formation, mitochondrial activity to CSE expression, and mitochondrial activity to nitrotyrosine formation. High *R*^2^ values might in some cases rather be due to inter-group differences than correlations between the parameters per se; thus, *R*^2^ values for (unpooled) separate groups are given in Additional file 1: Table S3

Kidney immunohistochemistry (see Fig. 3) revealed significantly increased levels of nitrotyrosine and extravasated albumin in sepsis (Fig. 3a, b), concomitant with reduced CSE and PGC1α expression (Fig. 3c, d). PGC1α expression, mitochondrial respiratory activity, and creatinine clearance correlated with CSE expression. There was an inverse relationship with nitrotyrosine formation and albumin extravasation. Finally, kidney nitrotyrosine formation was correlated with the degree of coupling of the mitochondrial respiratory chain (OxPhos/ETS to nitrotyrosine *p* = 0.002) and inversely correlated with maximum mitochondrial respiratory activity in the coupled state (OxPhos) (correlations depicted in Fig. 4).

## Discussion

We were able to confirm the initial hypothesis of an association between CSE expression and kidney dysfunction, mitochondrial activity, and oxidative/nitrosative stress in porcine septic AKI with underlying co-morbidity. The main findings of this study were (i) septic AKI accompanied by a marked drop in CSE expression which correlated with (ii) decreased glomerular filtration and (iii) reduced mitochondrial respiratory activity. Hyperlactatemia, metabolic acidosis, and enhanced glucose turnover were observed in the septic arm. O_2_ saturation and histopathological tissue injury did not differ between the sham and septic groups.

The lack of morphological changes associated with AKI, consistent with the literature [2, 3], led us to investigate renal protein expression. CSE is constitutively expressed in the kidney, and its expression correlates with the maintenance of glomerular filtration [1]. We were able to confirm these results; a sepsis-induced reduction of CSE expression was associated with kidney barrier dysfunction (indicated by the increased albumin extravasation; Fig. 3b, c) and thus contributed to the reduced glomerular filtration, as evinced in the impaired CrCl (Fig. 2a).

Furthermore, CSE has been reported to play a role in regulating glucose metabolism and mitochondrial function [8] through a PGC1α-dependent mechanism [12]. Consistent with these findings are reports that PGC1α expression in the kidney of septic animals also has been shown to be reduced in proportion to the severity of the impaired glomerular filtration rate [36]. This study was able to confirm a reduction of PGC1α expression concomitant with the loss of CSE in the septic kidney (Fig. 3d), which correlated with decreased mitochondrial respiratory activity (Fig. 2b). These findings are consistent with reports of reduced mitochondrial respiratory activity in septic patients and animal models of sepsis [7, 8]. Additionally, PGC1α has been shown to regulate mitochondrial oxidative metabolism, and its overexpression protected podocytes in renal injury preventing mitochondrial dysfunction [37, 38].

Nitrotyrosine formation is a marker of injury and oxidative and nitrosative stress and was found to be markedly elevated in the septic in contrast to the sham kidney (Fig. 3a). Impaired oxidative phosphorylation (OxPhos) in podocytes results in increased ROS production and functional alterations which may account for disruption of the glomerular filtration barrier and reduced function [39, 40]. The inverse correlation of nitrotyrosine formation with OxPhos (Fig. 4) implies a direct relationship of increased ROS/RNS with impaired mitochondrial function, as previously reported by others [41–43]. In turn, the linear correlation of OxPhos/ETS ratio to nitrotyrosine formation suggests that in sepsis, the mitochondria themselves are at least partly responsible for the increased ROS/RNS production, probably due to the strong coupling of the electron transfer system to ATP synthesis. Limited uncoupling can reduce tissue oxidative stress, whereas inhibiting the uncoupling leads to increased mitochondrial membrane potential with greater mitochondrial ROS production [44–46]. H_2_S can serve as a direct scavenger of ROS and peroxynitrite [47, 48], whereas cysteine is a key substrate for glutathione production, an important cellular antioxidant, and cysteine deficiency has been reported to be associated with increased oxidative stress [49]. This is reflected in the significant inverse relationship between CSE expression and nitrotyrosine formation observed in this study (Fig. 4).

The intersection of mitochondrial biogenesis, ROS generation, and inflammatory responses has been shown to be relevant in disease [50]. TNFα and IL-6 are recognized early players in the clinical response to sepsis and shown to be locally produced by intrinsic kidney cells in the early phase of injury [51, 52]. Elevated serum levels of IL-6 were shown to be indicators of animals that developed AKI, in a rat cecal ligation puncture model, in contrast to those that did not develop AKI, and the increased IL-6 preceded evidence of morphological kidney injury [53]. Our findings of elevated TNFα and IL-6 levels in sepsis-induced AKI (Additional file 1: Table S2) are consistent with these reports.

Mitochondrial dysfunction caused by or coinciding with inflammation may contribute to metabolic derangement of O_2_ utilization in septic AKI. Recent animal studies [2, 54] as well as the O_2_ saturation levels reported here (Additional file 1: Table S2) suggest that septic AKI is not necessarily due to the reduced tissue oxygen availability but rather due to the deranged cellular O_2_ metabolism, as suggested by the hyperlactatemia, metabolic acidosis, and reduced mitochondrial activity (Additional file 1: Table S2 and Fig. 2b). Recently, it has been shown that in sepsis, activated immune cells switch their cellular metabolism from oxidative phosphorylation to glycolysis (Warburg effect), even if oxygen is abundant [55, 56]. The reduced activity of complex II suggests the loss of mitochondrial “reserve capacity” in the kidneys of septic pigs in this study. Normally, mitochondria can make use of a “reserve capacity” in situations of increased energy demands by increasing complex II activity in response to enhanced glucose oxidation [57]. Hyperlactatemic patients suffering from septic shock have been reported to have increased lactate production concomitant to increased glucose turnover which is also reflected in this atherosclerotic septic pig model [58]. Interestingly, although the co-morbid septic pigs had increased glucose turnover overall, blood glucose levels did not differ from the sham group (Fig. 1), suggesting that the septic group was unable to mount an “adaptive” hyperglycemic stress response. This might be related to the reduced kidney CSE expression, as CSE^−/−^ mice have been previously shown to have reduced rates of gluconeogenesis [12].

At first glance, this might be counterintuitive, since in comparison to the septic group, the sham animals with maintained kidney CSE levels had lower glucose rate of appearance (Fig. 1 and 3c). However, these animals did not need to increase glucose turnover as a stress response and had very low rates of catecholamine administration compared to septic animals (Additional file 1: Table S1). Considering previous results from our group in a sepsis study in non-co-morbid young pigs, which displayed two times higher rates of glucose appearance [59], atherosclerotic pigs in this study seem to fail in their stress-related upregulation of gluconeogenesis. The kidney can account for 25–40% of all glucose released into the circulation and thus plays an important role in glucose homeostasis [6, 60, 61], especially under conditions of catecholamine treatment. Lactate is the predominant precursor for gluconeogenesis in healthy subjects stimulated with epinephrine to simulate physiological stress [60]. Increased lactate without hyperglycemia could be interpreted as a state wherein the body was no longer able to generate a hyperglycemic adaptive response to stress [6]. Pertinent to the current findings is the strong relation that was reported with regard to hyperlactatemia and “concomitant relative ‘normoglycemia’” and its association with the development of AKI [6]. Nonetheless, a limitation to the study is that we cannot discriminate how the higher norepinephrine infusion rates affected glucose metabolism, reduced CSE expression, and contributed to the more pronounced kidney dysfunction or if they simply reflected the severity of the disease in the individual animals.

## Conclusions

To the best of our knowledge, this is the first report of a relation between CSE expression, renal tissue mitochondrial function, and the severity of AKI in a clinically relevant resuscitated large animal model of polymicrobial sepsis. AKI was correlated with the reduced CSE and PGC1α expression, decreased mitochondrial respiratory activity concomitant with increased barrier dysfunction, increased oxidative stress, and metabolic acidosis which, combined, manifested in an inability of the cells to utilize oxygen [62]. Consequently, maintenance of CSE expression and endogenous H_2_S availability might attenuate sepsis-induced metabolic alterations [8, 20, 63].

## Additional file

Additional file 1: (DOCX 86 kb)

## Abbreviations

AKI: Acute kidney injury
CO_2_: Carbon dioxide
CrCl: Creatinine clearance
CSE: Cystathionine-γ-lyase
CVP: Central venous pressure
ETS: Electron transfer system
FBM: Familial hypercholesterolemia Bretoncelles Meishan
FCCP: Carbonyl cyanide-p-trifluoromethoxyphenylhydrazone
H_2_S: Hydrogen sulfide
HR: Heart rate
IL-6: Interleukin 6
MAP: Mean arterial pressure
NGAL: Neutrophil gelatinase-associated lipocalin
NO: Nitric oxide
NoA: Noradrenaline
OxPhos: Oxidative phosphorylation
PAOP: Pulmonary artery occlusion pressure
PEEP: Positive end-expiratory pressure
PGC1α: Peroxisome proliferator-activated receptor gamma coactivator 1-α
RNS: Reactive nitrogen species
ROS: Reactive oxygen species
TNFα: Tumor necrosis factor α
