# Supplementary material for: Cystathionine-γ-lyase expression is associated with mitochondrial respiration during sepsis-induced acute kidney injury in swine with atherosclerosis
Source: Intensive Care Med Exp. 2018 Oct 20;6:43. doi: 10.1186/s40635-018-0208-z (PMC6195873; doi:10.1186/s40635-018-0208-z)
Supplement: Supplementary file 1 — (DOCX 86 kb) [file 40635_2018_208_MOESM1_ESM.docx]

**Supplementary Material**

**Methods**

Immunoblotting for anti-cystathionine-γ-lyase (CSE; St John’s Laboratory, London, UK) was performed as described previously (Hartmann et al. 2018). Primary antibodies were detected by using horseradish peroxidase-conjugated secondary antibodies (Cell Signaling, Danvers, MA, USA or Santa Cruz, Dallas, TX, USA). Anti-β-actin (Santa Cruz, Dallas, TX, USA) served as a loading control. Densitometry measurements were performed using NIH Image J software (http://rsb.info.nih.gov/nih-image), results are presented as densitometric sum.

**Supplemental Tables**

**Table S1:** Systemic physiologic parameters, as previously published in [20].

|  |  | | baseline | 24 h | Ref. Value |
| --- | --- | --- | --- | --- | --- |
| NoA  (µg·kg^-1^·min^-1^)^*^ | Sham | n=5 | 0.06 (0.02; 0.13) | | n.a. |
|  | sepsis | n=6 | 1.23 (0.66; 3.26)^b^ | |  |
| Hemoglobin (g/dl) | sham | n=5 | 8.8 (8.6; 9.4) | 9.2 (9.1; 9.8) | 12.3-15.3 |
|  | sepsis | n=8 | 8.9 (8.5; 9.7) | 11.7 (11.1; 12.3)^a,b^ |  |
| Heart rate (bpm) | sham | n=5 | 88 (73; 104) | 102 (68; 115) | < 160 |
|  | sepsis | n=8 | 88 (74; 106) | 156 (140; 166)^a,b^ |  |
| Mean arterial pressure (mmHg) | sham | n=5 | 100 (90; 106) | 103 (94; 119) | max. +/- 10% of baseline |
|  | sepsis | n=8 | 103 (91; 112) | 65 (61; 81)^a,b^ |  |
| Central venous pressure (mmHg) | sham | n=5 | 8 (7; 13) | 10 (9; 15)^a^ | < 18 |
|  | sepsis | n=8 | 10 (6; 13) | 17 (14; 18)^a^ |  |
| Cardiac output (ml·kg^-1^·min^-1^) | sham | n=5 | 61 (52; 79) | 64 (42; 92) | n.a. |
|  | sepsis | n=8 | 64 (52; 69) | 87 (62; 130)^a^ |  |
| PaO_2_ (mmHg) | sham | n=5 | 158 (142; 180) | 159 (138;177) | n.a.^#^ |
|  | sepsis | n=8 | 170 (161; 183) | 93 (62; 155)^a,b^ |  |
| PaCO_2_ (mmHg) | sham | n=5 | 35 (35; 39) | 35 (33; 36) | 35-40 |
|  | sepsis | n=8 | 38 (34; 40) | 35 (32; 44) |  |
| arterial pH | sham | n=5 | 7.46 (7.44; 7.46) | 7.44 (7.43; 7.46) | 7.35-7.45 |
|  | sepsis | n=8 | 7.45 (7.43; 7.48) | 7.37 (7.19; 7.43)^a,b^ |  |
| Base excess (mmol/l) | sham | n=5 | 1.1 (0.8; 1.8) | -0.1(-1.45; 0.65) | -2.0 to +2.0 |
|  | sepsis | n=8 | 1.5 (0.4; 2.3) | -8.5 (-14.6; -3.7) ^a,b^ |  |
| Lactate (mmol/l) | sham | n=5 | 1.4 (1.0; 1.6) | 0.6 (0.6; 1.2) | < 2.0 |
|  | sepsis | n=8 | 0.8 (0.6; 1.5) | 6.1 (2.0; 10.7) ^a,b^ |  |

Data given as median (interquartile range). ^*^ NoA is administered based on mean arterial pressure (see Methods section) during the septic shock, ^#^ mechanical ventilation adjusted according to our previous work (if PaO_2_/FiO_2_ < 300mmHg = inspiratory/expiratory ratio 1:1, PEEP 12cm H_2_O; if PaO_2_/FiO_2_ < 200mmHg = PEEP 15cm H_2_O [10,19,26]), a p<0.05 in comparison to baseline, b p<0.05 in comparison to sham in two-way ANOVA

**Table S2:** Renal vein blood analysis, as previously published in [19,26].

|  |  | baseline | 24h peritonitis |
| --- | --- | --- | --- |
| O_2_ saturation (%) | sham | 83 (81; 83) | 83 (78; 84) |
|  | sepsis | 84 (78; 87) | 66 (45; 83) |
| pH | sham | 7.45 (7.44; 7.45) | 7.42 (7.42; 7.44) |
|  | sepsis | 7.42 (7.42; 7.44) | 7.28 (7.12; 7.36)^a,b^ |
| Base excess (mmol/l) | sham | 1.8 (1.8; 2.4) | 1.1 (0.7; 1.2) |
|  | sepsis | 1.8 (1.0; 3.1) | -4.9 (-12.0; -2.2)^a,b^ |
| Lactate (mmol/l) | sham | 1.1 (1.0; 1.2) | 1.0 (0.7; 1.2) |
|  | sepsis | 1.0 (0.7; 1.4) | 4.7 (2.5; 8.9)^a,b^ |
| IL6 (ng/g_protein_) | sham | 1.7 (1.3; 1.7) | 6.9 (1.7; 7.9) |
|  | sepsis | 2.0 (1.8; 2.1) | 1153.4 (236.4; 2002.5)^a,b^ |
| TNFα (ng/g_protein_) | sham | 0.8 (0.6; 1.2) | 1.6 (1.3; 2.0) |
|  | sepsis | 0.8 (0.7; 1.2) | 15.3 (5.9; 21.7)^a,b^ |

Data given as median (interquartile range). a p<0.05 in comparison to baseline, b p<0.05 in comparison to sham in two-way ANOVA

**Table S3:** R^2^ values for correlations with (unpooled) separate groups, respectively.

|  | CrCl  (ml/min) | | OxPhos  jO_2_ (pmol·s^-1^·mg^-1^) | | PGC1α expression | | albumin extravasation | | nitrotyrosine formation | |
| --- | --- | --- | --- | --- | --- | --- | --- | --- | --- | --- |
|  | sham | sepsis | sham | sepsis | sham | sepsis | sham | sepsis | sham | sepsis |
| NoA infusion  (µg·kg^-1^·min^-1^) | 0.36 | 0.49 | n.a. | 1 |  |  |  |  |  |  |
| OxPhos  jO_2_ (pmol·s^-1^·mg^-1^) | n.a. | 0.89 |  |  |  |  |  |  |  |  |
| CSE expression | 0.86 | 0.20 | 0.24 | 0.62 | 0.57 | 0.09 | 0.73 | 0.02 | 0.02 | 0.47 |
| nitrotyrosine formation |  |  | 0.85 | 0.84 |  |  |  |  |  |  |

**Supplemental Figure S1**


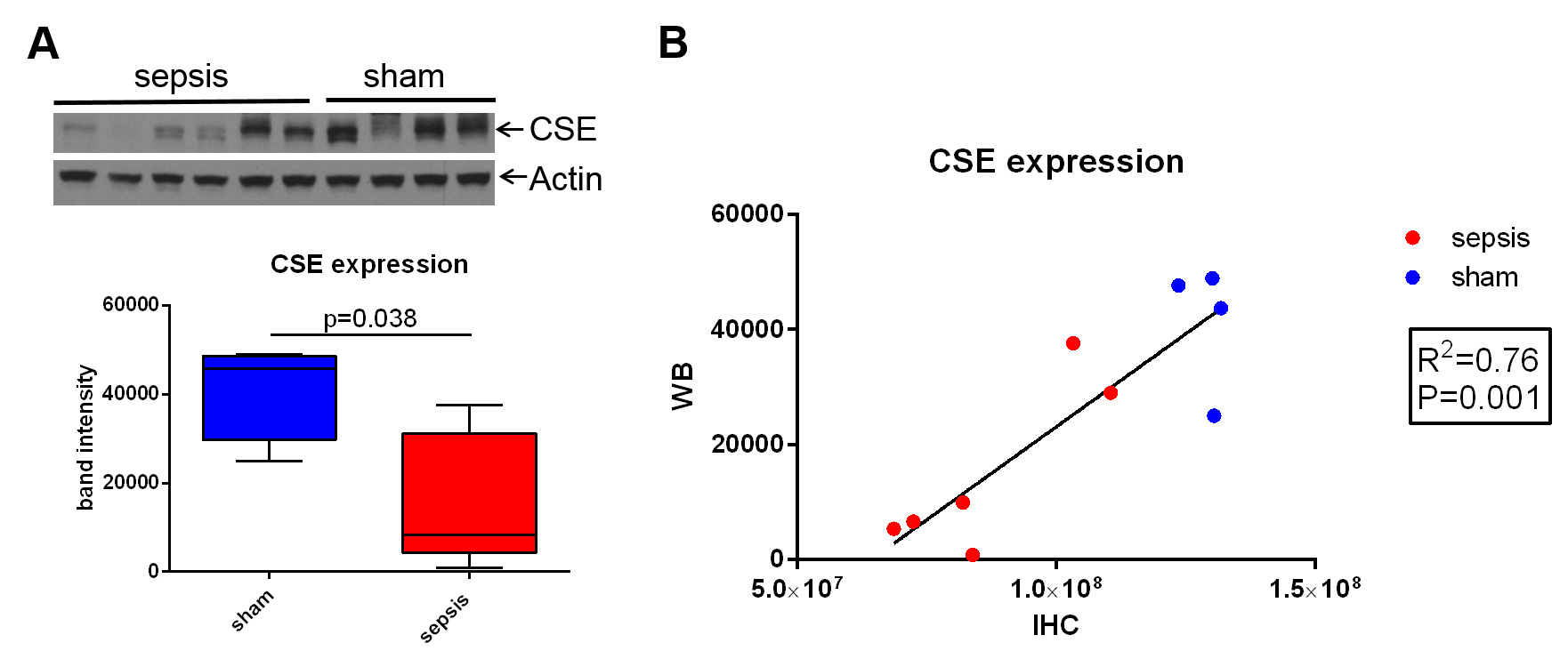


**Figure S1:** Kidney CSE protein expression levels for sham vs. sepsis, detected by western blot (WB, **A**) correlate with kidney CSE expression levels detected by immunohistochemistry (IHC, **B**).

**References**

Hartmann C, Gröger M, Noirhomme JP, Scheuerle A, Möller P, Wachter U, Huber-Lang M Nussbaum B, Jung B, Merz T, McCook O, Kress S, Stahl B, Calzia E, Georgieff M, Radermacher P, Wepler M. In-Depth Characterization of the Effects of Cigarette Smoke Exposure on the Acute Trauma Response and Hemorrhage in Mice. Shock. 2018. Volume Publish Ahead of Print.
